# Examining Outcomes and Challenges of Telepsychiatry in Australian Elderly: A Scoping Review

**DOI:** 10.1155/2023/8864591

**Published:** 2023-10-17

**Authors:** Sodabeh Abazari, Khadijeh Moulaei, Manoj George

**Affiliations:** ^1^Older Persons Mental Health Service, West Moreton Health, Ipswich, Queensland, Australia; ^2^Department of Health Information Technology, Faculty of Paramedical, Ilam University of Medical Sciences, Ilam, Iran

## Abstract

**Methods:**

To find relevant articles, we searched PubMed, Scopus, and Web of Science databases. We used a data extraction form to gather information from primary studies. Two researchers followed inclusion and exclusion criteria to select studies and extract data. Disagreements were resolved through discussion with all researchers. Studies needed to be in English, about telepsychiatry for Australian seniors, and use any technology type (synchronous, asynchronous, or both). We excluded nontelepsychiatry articles, books, book chapters, conference abstracts, and editor letters.

**Results:**

Telepsychiatry was effectively employed to manage depression, anxiety, delirium, and cognitive impairments. Among these four disorders, telepsychiatry was mostly used for depression. Videoconference and telephone were mostly used to provide telepsychiatry services. Most telepsychiatry services for Australian seniors included “patient education on disorder control and management,” “creating continuous interaction between the patient and the therapist,” and “remote patients' assessment.” “Reductions in symptoms of disorders,” “improving patients' satisfaction with telepsychiatry,” and “cost-effectiveness of telepsychiatry” were the most important positive outcomes of using telepsychiatry. We also identified four challenges in using telepsychiatry for elderly individuals in Australia.

**Conclusions:**

This study is the first scoping review in Australia and provides valuable insight into telepsychiatry for elderly individuals.

## 1. Introduction

Mental disorders impact thoughts, emotions, behaviors, and overall well-being, often causing distress and functional impairment [1]. Depression involves persistent sadness, low energy, and diminished interest, affecting sleep, appetite, and self-esteem [2]. Anxiety disorders manifest as excessive worry or fear, leading to physical symptoms such as an increased heart rate [3]. Moreover, various disorders exist, including bipolar disorder, schizophrenia, obsessive-compulsive disorder (OCD), and posttraumatic stress disorder (PTSD), each with unique symptoms and treatment approaches, often requiring therapy, medication, and lifestyle changes for effective management [4]. Mental disorders are spreading rapidly in different countries. Based on the Australian Bureau of Statistics (ABS) National Survey of Mental Health and Wellbeing, a significant proportion of Australians aged 16–85 (43.7% or 8.6 million) have experienced mental disorders in their lives, with 21.4% (4.2 million) encountering them within a year [5].

Mental disorders are recognized as the main cause of disability and disease burden throughout lifespan. The rapid growth of older and very elderly populations worldwide emphasizes the need for a clear understanding of how mental disorders manifest in older people, as the presence of mental disorders in this people has important implications for service delivery and public health policies [6]. There is considerable evidence of increased morbidity, mortality, hospitalisation, and loss of functional condition associated with common mental disorders in older individuals [7]. In different countries, including Australia, older people face many challenges and tensions and often have complex needs related to their mental health. Presence of chronic diseases, creating useless feelings, depression, hopelessness, loss of independence, loneliness, and social isolation are among the important threatening challenges caused by disorders related to the mental health of older people. In addition, mental disorders and challenges can reduce the quality of life, job loss, low self-confidence, anxiety, alcohol abuse, negative emotions, sleep disorders, and suicide in older individuals [8]. Therefore, if these disorders and challenges are not properly controlled and managed, older people will face irreparable problems. Moreover, numerous studies [9–11] have highlighted the persistent stigma and discrimination that individuals with mental disorders face in various aspects of their lives, including social interactions, employment opportunities, and access to healthcare. Lopresti et al. [12] also mentioned that lifestyle factors, including diet, exercise, and sleep, play a crucial role in mental health. Patients with mental disorders often struggle to adopt and maintain healthy lifestyle habits that could have a positive impact on their condition. One of the ways to help control and manage these disorders is the use of telepsychiatry.

Telepsychiatry has been used successfully in rural South Australia, especially to assess patients whose problems and challenges were considered too urgent to wait for the next monthly psychiatrist visit [13]. Telepsychiatry refers to the delivery of mental health services via videoconferencing and has great potential to address mental health disparities by increasing the reach of mental healthcare to those living in rural regions or those with limited access to care [14]. Telepsychiatry can save money by providing early intervention, reducing costs by limiting medical trips, continuity, and better coordination of care [15]. In addition, some studies [14] have shown that the use of telepsychiatry can reduce lateness and no-show phenomena, as well as shorten the actual meeting time, thereby reducing crowding and waiting time bottlenecks. Li et al. [16] also noted that telepsychiatry can, in addition to compensating for the shortage of health professionals, enable providers in a hospital setting to complete virtual psychiatric assessments, provide teletherapy, communicate briefly and check in with patients, and provide patient education.

To our knowledge, other review studies have focused on the identification of telepsychiatry outcomes [14], the evaluation of the effectiveness of videoconferencing in the management of mental illnesses [17], and developments in the area of telepsychiatry [18]. In this scoping review, various services provided through telepsychiatry for elderly individuals in Australia are identified. Moreover, this study focuses on identifying the outcomes and challenges of using telepsychiatry by elderly individuals in Australia.

## 2. Materials and Methods

The present study is a scoping review that examines the positive and challenges of utilizing telepsychiatry among elderly individuals in Australia. In order to select the relevant studies and report the extracted results, we followed the PRISMA scope review checklist.

### 2.1. Information Sources and Search Strategy

Three databases, namely, PubMed, Web of Science, and Scopus, were searched for articles focusing on telepsychiatry in Australian geriatrics. The search was conducted up to 1 December 2022. The following keywords and search strategy were employed to carry out the search process: ((Telepsychiatry OR Tele-psychiatry OR telemental OR telemedicine OR telehealth OR teleconsultation OR tele-consultation OR remote consultation) AND (ageing people OR older people OR older patient OR older subject OR older age OR older man OR older men OR older male OR older woman OR older women OR older female OR older population OR older person) AND (Australia)).

The search process was conducted in all three databases in December 2022. It is noteworthy that no limitations were imposed on the starting point of the database search.

### 2.2. Eligibility Criteria

In this study, articles focusing on telepsychiatry and Australian geriatrics were included. The inclusion criteria comprised articles written in English that addressed any mental health disorder, specifically targeting Australian older individuals aged 60 years or older. Both synchronous (real-time or live video) and asynchronous (stored-and-forward) telepsychiatry approaches were considered. Exclusion criteria encompassed articles that did not specifically concentrate on telepsychiatry in elderly individuals in Australia. Furthermore, books, book chapters, conference abstracts, and letters to the editor were excluded from the analysis.

According to the World Health Organization (WHO), individuals aged 60 years and older are classified as the elderly population. Accordingly, this study specifically included studies in which the participants were 60 years old and/or older [19].

### 2.3. Selection Sources of Evidence and Data Extraction

In the initial step, all articles were retrieved and imported into EndNote software. Duplicate articles were subsequently excluded by the researcher. In the subsequent stage, articles were selected based on their title, abstract, and adherence to the inclusion and exclusion criteria. All eligible articles were thoroughly reviewed and studied, and final approval was obtained. In the event of any disagreements, the study researchers collectively deliberated to reach a consensus on each article. Once the articles were approved, their full text was meticulously examined and analyzed to extract the necessary information.

### 2.4. Data Charting Process and Data Items

A data extraction form was developed and utilized to extract information from the validated studies. The form underwent validation by two medical informatics experts experienced in designing telemedicine systems and a psychiatrist. The data extraction form encompasses fields such as publication years, study types, study aim, the number of participants, mental disorder, types of telepsychiatry intervention, follow-up duration, types of services (asynchronous or synchronous), outcomes, and challenges. In cases where articles had missing data, correspondence was established with the corresponding authors via email to request the required information. Ultimately, the extracted data were imported into an Excel file.

### 2.5. Synthesis of Results

After importing the data into an Excel file, we classified them qualitatively and reported the results, frequencies, and percentages. To synthesize the data, we used the advancement scoring method recommended by Levac et al. [20]. One of the authors conducted a data review, which involved checking for spellings, cell formatting, and ensuring accurate calculations and analyses in Excel. Descriptive analyses, such as calculating frequencies and percentages, were performed on the extracted data from the studies since scoping reviews do not aim to summarize or weigh evidence from different studies [21]. The descriptive data and findings from the reviewed articles were then presented in tables and figures, aligning with the study's objectives, to effectively communicate the review's findings.

## 3. Results

### 3.1. Selection of Sources of Evidence

**Figure 1:**
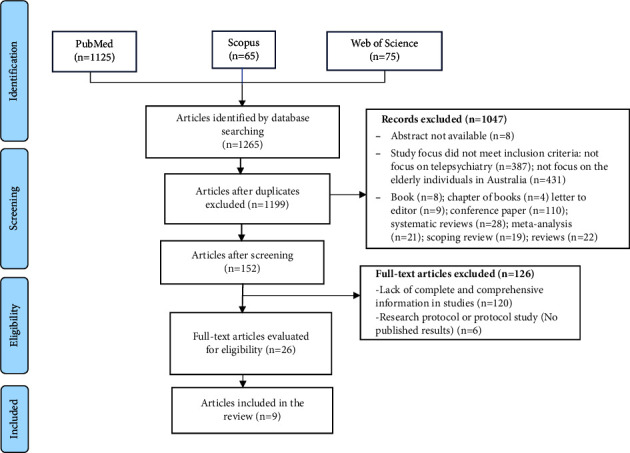
Study selection process.

In total, 1,265 articles were retrieved. After excluding duplicates, the remaining 1,199 studies were carefully reviewed and evaluated based on inclusion and exclusion criteria. Finally, nine articles were included in the study (more details in Figure 1).

### 3.2. Characteristics of the Included Studies

An overview of selected articles is presented in Table 1.

**Figure 2:**
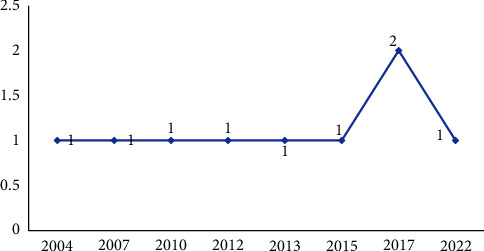
Distribution of the studies in terms of year of publication.

Most of the studies conducted were randomized clinical trial (RCTs) (*n* = 5) [22, 25–28] (more details in Table 1). Moreover, as Figure 2 shows, the first study of telepsychiatry in elderly individuals in Australia was published in 2004.

**Figure 3:**
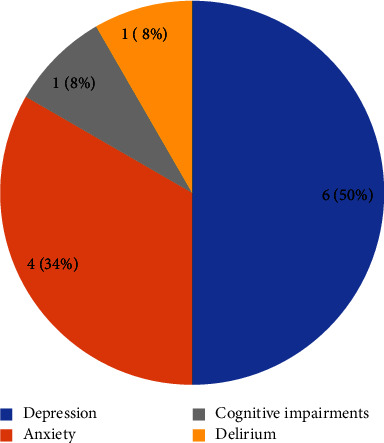
The distribution of the studies based on mental disorders. Note: in some studies, more than one disorder was reported.

Telepsychiatry was utilized for the treatment, control, or management of four types of mental disorders in elderly individuals in Australia: depression, anxiety, delirium, and cognitive impairments. Out of these four disorders, telepsychiatry was predominantly used for depression (*n* = 6, 50%) (as shown in Figure 3).

The telepsychiatry services for elderly individuals in Australia were provided through videoconferencing, telephone, and web-based programs. Videoconference (*n* = 4, 44%) [22–24, 30] and telephone (*n* = 4, 44%) [25, 27–29] were mostly used to provide telepsychiatry services to Australian older people. Also, telepsychiatry services were provided in asynchronous and synchronous forms. Most of the studies used synchronous form to provide these services (*n* = 8, 88%) [22–25, 27–30] (more details in Table 1).

### 3.3. Services Provided with Telepsychiatry for Elderly Individuals in Australia

Table 2 displays the various telepsychiatry services for elderly individuals in Australia, encompassing six different types of services. Among them, the most notable services were “patient education on disorder control and management,” “creating continuous interaction between the patient and the therapist,” and “remote patient assessment.”

### 3.4. Outcomes and Challenges of Using Telepsychiatry for Elderly Individuals in Australia

The outcomes of using telepsychiatry for elderly individuals in Australia are presented in Table 3. Among the nine identified outcomes, “reductions in symptoms of disorders,” “improving patients' satisfaction with telepsychiatry,” and “cost-effectiveness of telepsychiatry” were the most important. It should be noted that no negative outcome was reported in any of the studies.

Moreover, several challenges have been identified in the use of telepsychiatry for elderly persons Australian, including overall quality of the voice, hearing and difficulties in understanding accents during interactions between patients and physicians, low bandwidth, the inability to perform physical examinations during telepsychiatry [22], and the inefficiency of telepsychiatry services for patients with visual or sensory disorders [23].

## 4. Discussion

Telepsychiatry refers to the practice of providing psychiatric evaluation, consultation, and treatment remotely, utilizing telecommunications technology such as videoconferencing and phone calls. This approach allows mental health professionals to connect with patients regardless of their geographical location, overcoming barriers to access and enhancing the reach of mental health services [14]. Telepsychiatry presents substantial possibilities in elderly care, particularly for those hindered by mobility constraints or geographical distance from healthcare centers. By utilizing this technology, mental health experts can provide tailored evaluations, therapeutic measures, and continuous assistance to seniors in the convenience of their residences [31]. In our scoping review, services provided with telepsychiatry for elderly individuals in Australia and outcomes and challenges of using telepsychiatry for these people were identified. This study shows that telepsychiatry services were provided to the elderly individuals through videoconference, telephone, and web-based programs. “Patient education on disorder control and management,” “creating continuous interaction between the patient and the therapist,” and “remote patients' assessment” were the most common services provided through telepsychiatry. We also identified nine outcomes and four challenges of using telepsychiatry for Australian older people.

“Reductions in symptoms of disorders,” “improving patients' satisfaction with the telepsychiatry,” and “cost-effectiveness of telepsychiatry” were the most common positive outcomes of using telepsychiatry for elderly individuals in Australia in our review. There is considerable evidence for the use of telepsychiatry as an efficient method of providing psychiatric services. Hubley et al. [14] reviewed 134 articles in a review study and concluded that telepsychiatry can improve patient satisfaction, quality of care, and cost-effectiveness. Moreover, geriatric psychiatry provides an opportunity to expand mental health services to older adults who are difficult to reach face-to-face, as well as to improve the overall continuum of care [32]. By analyzing 333 completed surveys of psychotherapists and psychiatrists, Mishkin et al. [33] concluded that most participants felt that telepsychiatry was an acceptable practice—even for those working primarily with medical patient populations. In other words, there were positive ratings of completeness, overall satisfaction, and patient-reported satisfaction. Vadlamani et al. [34] also pointed out that telepsychiatry can effectively manage and control mental disorders and diseases, reduce treatment costs, increase patient and healthcare provider satisfaction, lead to an overall improvement in the quality of care, offer self-management training to patients, and provide equitable access to psychiatric services for patients in various regions. Findings from Rabinowitz et al.'s study [35] showed that at least more than $13,000 could be saved by telepsychiatry providing nursing home consultations if nursing home residents were transferred to a psychiatrist's office for treatment consultation. If the doctor visits the patient, maximum cost savings can be more than $232,000. In addition, more than 35 days of travel time can be avoided. By reducing (or removing) physician travel time, telepsychiatry allows psychiatrists to see more patients (either telepsychiatry or in-person) all day, which is a significant advantage in some locations that there is a shortage of physician. Further cost savings will be possible in situations where other more costly types of medical specialty care (e.g., dermatology and follow-up visits after surgery) could be provided by telehealth, assisting to avoid higher physician hourly costs.

However, it should be stated that the designers of telepsychiatry systems, the owners of these systems, policymakers, and managers of medical centers and hospitals should plan in such a way that the positive outcomes of the use of these systems by elderly individuals are maximized, while negative outcomes are greatly reduced. It seems that training older people to use telepsychiatry efficiently and prevent technical problems can lead to positive outcomes. Mishkin et al. [33] pointed out that educating patients in telepsychiatry and preventing technical problems during online sessions are much more important and valuable than clinician telepsychiatry experience. This means that hospitals and health centers that invest in good training and technical support are likely to see positive outcomes for both patients and physicians, and these services should be proactively invested in building technological capacity. Ease of use or usability of telepsychiatry can also lead to beneficial and positive outcomes for patients and therapists. The National Institute of Standards and Technology (NIST) and the US Agency for Healthcare Research and Quality have both emphasized the need to measure and improve health information technology usability [36]. Usability is considered an essential component influencing the acceptance of health systems by older people. A usable health system with an age-friendly interface has many advantages for older individuals, including raising their well-being, reducing the risk of harm, and increasing accessibility [37]. In addition, the effective design and implementation of health systems for patients and health providers can provide effective, safe, efficient, and timely access to healthcare services [36].

Despite many patient concerns about confidentiality, privacy, and limited capacity to respond to psychiatric emergencies, we did not identify any published reports of these side effects in the use of telepsychiatry. In this review, we identified challenges such as overall quality of the voice, hearing and difficulties in understanding accents during interactions between patients and physicians, low bandwidth, the inability to perform physical examinations during telepsychiatry, and the inefficiency of telepsychiatry services for patients with visual or sensory disorders. Rout et al. [38] pointed out that restrictions in physical examination, agreeing in the “being there” element, and the ability of older individuals to use modern technologies, and guaranteeing privacy during the consultation are important challenges. There are also health insurance and medicolegal challenges. It has also been reported that older adults with disabilities or chronic diseases face multiple challenges when dealing with technology, such as altered cognition, vision and hearing problems, lack of trust, and privacy concerns [39]. It must be said that without adopting these skills and solving these problems, older people may not receive the benefits of health information and communication technologies (HICTs) in routine care. Hart et al. [40] found that older people need skills to accept and continuously use HICTs. So it is necessary to identify the factors of health information and communication technology use in order to facilitate the design of mitigation strategies to overcome the challenges of use [39]. Some of these limitations can be overcome through optimizing the design of ICT interventions such as increasing screen contrast to reduce visual acuity loss or greater use of audio technologies for people with visual impairments. Moreover, given the limitations of telemedicine physical examination, the Center for Telemedicine, National Institute of Mental Health and Neurosciences (NIMHANS), Bengaluru, India, developed a clinically oriented concept of “virtual physical examination” (VPE) exclusively for videoconferencing consultation where physicians can perform the inspection part of the systemic physical examination well [32].

Therefore, it should be noted that, despite the fact that telepsychiatry can bring many benefits to older individuals, its challenges should not be neglected and it is always looking for ways to overcome these challenges. With the advancement of technologies based on artificial intelligence, machine learning, speech recognition, robotics, Internet of things, etc., solutions can be provided to overcome these problems. For example, speech recognition technology can recognize speech, interpret it, and convert it into text. Speech to text can be very helpful for hearing impaired people. Or as another example, the availability of artificial intelligence tools may create more opportunities for risk identification and diagnostic services, especially in cases of shortage of health professionals, distance, and patient mobility problems.

In the end, it should be noted that the evolving field of telepsychiatry presents promising research avenues highlighted in this review. It suggests exploring how telepsychiatry affects elderly individuals with different mental disorders through neurobiological and psychological mechanisms, offering insights into symptom reduction and patient satisfaction. Longitudinal studies could reveal lasting effects on mental health for Australian elderly, especially in rural areas. Tailoring telepsychiatry to diverse elderly populations could optimize mental health service delivery. Policy implications call for integrating telepsychiatry in mental health frameworks for the elderly, backed by training for professionals to enhance remote care quality. Continued research is emphasized, urging funding for nuanced telepsychiatry studies. Rigorous investigations are needed to customize interventions for unique elderly needs, and comparative studies could support telepsychiatry's integration. Collaboration between mental health experts, technologists, and geriatric specialists could lead to innovative solutions. Practitioners must be trained in telepsychiatry techniques to balance technology with empathetic care. Ongoing collaboration between researchers, policymakers, and practitioners is crucial in utilizing telepsychiatry's potential to enhance mental healthcare for elderly Australians.

## 5. Strengths and Limitations of the Study

The strengths of the article entitled “Examining Outcomes and Challenges of Telepsychiatry in Australian Elderly: A Scoping Review” lie in its comprehensive exploration of telepsychiatry's utilization in addressing mental health challenges among elderly Australians. The study's unique contribution as the first scoping review in Australia highlights its pioneering nature in examining this specific context. The research effectively identifies a range of services offered through telepsychiatry, focusing on depression, anxiety, delirium, and cognitive impairments, and provides a thorough analysis of the outcomes and challenges associated with these telepsychiatry systems. By uncovering the positive outcomes such as symptom reduction, enhanced patient satisfaction, and cost-effectiveness, the article provides valuable insights into the potential benefits of telepsychiatry for the elderly population. Moreover, the identification of challenges specific to using telepsychiatry for elderly individuals in Australia underscores the study's depth of analysis and real-world applicability. Overall, the article contributes significantly to the understanding of the telepsychiatry's role in managing mental health issues in the elderly, making it a noteworthy addition to the literature in the field.

In this study, we had several limitations. In this study, we only reviewed studies in English and studies related to elderly individuals in Australia. If a study was published in a language other than English, or related to older people from other countries, we did not review it. It is suggested considering these limitations in other studies. Also, only three databases, Scopus, PubMed and Web of Science, were searched to find the required studies. Future studies by searching more databases can achieve more comprehensive results. We also did not focus on critical appraisal of individual sources in this review. Although this section is optional according to the PRISMA scope review checklist, it can be considered in future studies. Moreover, the review incorporates studies with a limited sample size, potentially compromising the statistical power and generalizability of findings. To address this, future research could emphasize larger and more diverse participant groups to enhance the robustness and applicability of the conclusions drawn.

## 6. Conclusion

This scoping review identified various telepsychiatry services available for elderly individuals in Australia as well as the positive outcomes and general challenges associated with their use. The review found that telepsychiatry is a valuable tool in the treatment of mental disorders and has the potential to become an efficient way to provide high-quality psychiatric care. Telepsychiatry appears especially beneficial for patients in isolated locations, allowing them to access specialist mental healthcare with greater ease and cost-effectiveness. Telepsychiatry also helps distribute psychiatric services more equitably throughout the world or a particular country. It is worth mentioning that this study provides a foundation of knowledge on the use of telepsychiatry, not just for elderly individuals in Australia but also for older populations in other countries with similar technology and demographic characteristics.

In conclusion, telepsychiatry is a promising approach to providing psychiatric services to elderly individuals in Australia. The positive outcomes and cost-effectiveness of telepsychiatry make it an attractive option for healthcare providers, policymakers, and patients. However, it is crucial to address the challenges associated with its use, including patient education and technical support, to ensure that telepsychiatry delivers desired outcomes. Investing in user-friendly technology, support services, and policy reform can enhance the effectiveness of telemedicine services and ensure timely access to healthcare services for older people.

## Figures and Tables

**Table 1:** Overview of the included studies.

Ref	Study type	Study aim	Number of participants	Mental disorder	Type of intervention in telepsychiatry	Follow-up duration	Types of services	
							Asynchronous	Synchronous
[22]	Randomized clinical trial (RCT)	Evaluating the condition of the older people with dementia and depression through telehealth as compared to face-to-face administration	20	Delirium and depression	Videoconference	Six weeks		✓
[23]	No data available	Comparison of video conferencing and face-to-face evaluations in the diagnosis of Alzheimer's disease in the older individuals with cognitive impairment and depression	20	Cognitive impairments and depression	Videoconference	One week		✓
[24]	Pilot study	Investigating the acceptability and effectiveness of a brief behavioral activation therapy through videoconferencing to the older people with depressive disorder	3	Depression	Videoconference	1-month		✓
[25]	RCT	Investigating the effectiveness of a cognitive-behavioral therapy program provided through the internet in the older individuals with anxiety	22	Anxiety	Telephone	Three-month		✓
[26]	RCT	Evaluating the effects of internet-delivered cognitive behavioral therapy (iCBT) on the severity of depressive symptoms and adherence to physician recommendations and lifestyle interventions in the older with depression and high risks of cardiovascular disease	487	Depression	Web-based program	12 weeks	✓	✓
[27]	RCT	Investigating the outcomes of telephone-delivered cognitive-behavioral therapy compared to indirect telephone-based supportive therapy in rural older adults with anxiety disorders	141	Anxiety	Telephone	4 months		✓
[28]	RCT	Investigating the effects of cognitive behavioral therapy and social control provided over the phone on anxiety and depression symptoms in people with chronic obstructive pulmonary disease	110	Depression and anxiety	Telephone	8-week		✓
[29]	Case study	Investigating the effects of using a friend over the phone in a chronic obstructive pulmonary disease patient with severe anxiety and depression	No data available	Anxiety	Telephone	8 weeks		✓
[30]	Cross-sectional study	Investigating the effects of video-interpreting and face-to-face interpretation on dementia and depression in the older people	45	Depression	Videoconference	7–14 days		✓

**Table 2:** Telepsychiatry services for elderly individuals in Australia.

Services	References	Services frequency based on the number of references
(1) Patient education on disorder control and management	[24–29]	6
(2) Creating continuous interaction between the patient and the therapist	[22–24, 26, 30]	5
(3) Remote patient assessment	[22, 23, 30]	3
(4) Providing remote counseling data and social support services	[22]	1
(5) Diagnosis of mental disorders	[22]	1
(6) Continuous monitoring of patients	[29]	1

**Table 3:** Outcomes of using telepsychiatry for elderly individuals in Australia.

Outcomes	References	Outcome frequency based on the number of references
(1) Reductions in symptoms of disorders	[24–29]	6
(2) Improving patients' satisfaction with telepsychiatry	[24, 25, 28]	3
(3) Cost-effectiveness of telepsychiatry	[25, 28, 30]	3
(4) Quick and easy diagnosis of mental disorders	[22, 23]	2
(5) Increasing adherence to treatment processes	[25, 26]	2
(6) Reliability of telepsychiatry systems	[24, 30]	2
(7) Increasing access to treatment services	[24]	1
(8) No adverse effect on patients	[26]	1
(9) Significant improvement in general self-efficacy	[28]	1

## Data Availability

All data generated or analyzed during this study are included in this published article.
